# Patient satisfaction and value based purchasing in hospitals, Odisha, India

**DOI:** 10.2471/BLT.24.290519

**Published:** 2024-06-04

**Authors:** Liana Woskie, Anuska Kalita, Bijetri Bose, Arpita Chakraborty, Kirti Gupta, Winnie Yip

**Affiliations:** a Department of Community Health, Tufts University, 574 Boston Ave, Suite 208, Medford MA 02155, United States of America (USA).; b Department of Global Health and Population, Harvard University, Boston, USA.; c Oxford Policy Management India Pvt Ltd, New Delhi, India.

## Abstract

**Objective:**

To examine how a general inpatient satisfaction survey functions as a hospital performance measure.

**Methods:**

We conducted a mixed-methods pilot study of the Hospital Consumer Assessment of Health Providers and Systems survey in Odisha, India. We divided the study into three steps: cognitive testing of the survey, item testing with exploratory factor analysis and content validity indexing. Cognitive testing involved 50 participants discussing their interpretation of survey items. The survey was then administered to 507 inpatients across five public hospitals in Odisha, followed by exploratory factor analysis. Finally, we interviewed 15 individuals to evaluate the content validity of the survey items.

**Findings:**

Cognitive testing revealed that six out of 18 survey questions were not consistently understood within the Odisha inpatient setting, highlighting issues around responsibilities for care. Exploratory factor analysis identified a six-factor structure explaining 66.7% of the variance. Regression models showed that interpersonal care from doctors and nurses had the strongest association with overall satisfaction. An assessment of differential item functioning revealed that patients with a socially marginalized caste reported higher disrespectful care, though this did not translate into differences in reported satisfaction. Content validity indexing suggested that discordance between experiences of disrespectful care and satisfaction ratings might be due to low patient expectations.

**Conclusion:**

Using satisfaction ratings without nuanced approaches in value-based purchasing programmes may mask poor-quality interpersonal services, particularly for historically marginalized patients. Surveys should be designed to accurately capture true levels of dissatisfaction, ensuring that patient concerns are not hidden.

## Résumé

**Objectif:**

Examiner le fonctionnement d’une enquête générale de satisfaction des patients hospitalisés en tant que mesure de la performance des hôpitaux.

**Méthodes:**

Nous avons mené une étude pilote mixte de l’enquête Hospital Consumer Assessment of Health care Providers and Systems à Odisha, en Inde. Nous avons subdivisé l’étude en trois étapes: tests cognitifs de l’enquête, test par items avec analyse factorielle exploratoire et indexation de la validité du contenu. Les tests cognitifs concernaient 50 personnes, qui ont discuté de leur interprétation des questions de l’enquête. L’enquête a ensuite été soumise à 507 patients hospitalisés dans cinq hôpitaux publics d’Odisha, puis a fait l’objet d’une analyse factorielle exploratoire. Enfin, nous avons interrogé 15 personnes pour évaluer la validité du contenu des questions de l’enquête.

**Résultats:**

Les tests cognitifs ont révélé que six des 18 questions de l’enquête n’étaient pas toujours comprises par les patients hospitalisés à Odisha, ce qui met en évidence les problèmes liés aux responsabilités en matière de soins. Une analyse factorielle exploratoire a permis d’identifier une structure à six facteurs expliquant 66,7% de la variance. Des modèles de régression ont mis en évidence que les soins interpersonnels prodigués par des médecins et des infirmières avaient le plus grand impact sur la satisfaction globale. Une évaluation du fonctionnement différentiel des items a révélé que les patients appartenant à une caste socialement marginalisée signalaient davantage d’irrespect dans les soins, bien que cela ne se traduise pas par des différences au niveau de la satisfaction déclarée. L’indexation de la validité du contenu a suggéré que la discordance entre les expériences d’irrespect dans les soins et les évaluations de satisfaction pourrait être due à la faiblesse des attentes des patients.

**Conclusion:**

L’utilisation d’évaluations de la satisfaction sans approches nuancées dans les programmes d’achat basés sur la valeur est susceptible de masquer des services interpersonnels de mauvaise qualité, en particulier pour les patients historiquement marginalisés. Les enquêtes doivent être conçues de manière à saisir avec précision les véritables niveaux d’insatisfaction, en évitant de masquer les préoccupations des patients.

## Resumen

**Objetivo:**

Examinar el funcionamiento de una encuesta general de satisfacción de los pacientes hospitalizados como medida de rendimiento de los hospitales.

**Métodos:**

Se realizó un estudio piloto de métodos mixtos de la encuesta de Evaluación del consumidor hospitalario sobre proveedores y sistemas de atención sanitaria en Odisha (India). Se dividió el estudio en tres pasos: prueba cognitiva de la encuesta, prueba de elementos con análisis factorial exploratorio e indexación de la validez del contenido. La prueba cognitiva consistió en que 50 participantes discutieran su interpretación de los elementos de la encuesta. A continuación, se administró la encuesta a 507 pacientes ingresados en cinco hospitales públicos de Odisha, tras lo cual se realizó un análisis factorial exploratorio. Por último, se entrevistó a 15 personas para evaluar la validez de contenido de los elementos de la encuesta.

**Resultados:**

Las pruebas cognitivas revelaron que seis de las 18 preguntas de la encuesta no se comprendían de forma coherente en el entorno hospitalario de Odisha, lo que evidenciaba problemas relacionados con las responsabilidades de la atención. El análisis factorial exploratorio identificó una estructura de seis factores que explicaban el 66,7% de la varianza. Los modelos de regresión mostraron que la atención interpersonal por parte de médicos y personal de enfermería presentaba la mayor asociación con la satisfacción general. Una evaluación del funcionamiento diferencial de los elementos reveló que los pacientes de una casta socialmente marginada informaron de una atención más irrespetuosa, aunque esto no se reflejó en diferencias en la satisfacción declarada. La indexación de la validez de contenido sugirió que la discordancia entre las experiencias de atención irrespetuosa y los índices de satisfacción podría deberse a las bajas expectativas de los pacientes.

**Conclusión:**

El uso de índices de satisfacción sin enfoques matizados en los programas de compras basadas en el valor puede enmascarar servicios interpersonales de mala calidad, en particular para pacientes históricamente marginados. Las encuestas deben diseñarse para captar con precisión los verdaderos niveles de insatisfacción, de forma que no se oculten las preocupaciones de los pacientes.

## ملخص

**الغرض:**

فحص كيفية عمل المسح العام لرضا المرضى في العيادات الداخلية كمقياس لأداء المستشفى.

**الطريقة:**

قمنا بإجراء دراسة تجريبية مختلطة الأساليب لمسح تقييم المستهلك في المستشفيات لمقدمي الرعاية الصحية والأنظمة في أوديشا، الهند. كما قمنا بتقسيم الدراسة إلى ثلاث خطوات: الاختبار الإدراكي للمسح، واختبار العناصر مع تحليل العوامل الاستكشافية، وفهرسة صلاحية المحتوى. شمل الاختبار الإدراكي 50 مشاركًا يناقشون تفسيرهم لبنود المسح. تم بعد ذلك إجراء المسح على 507 مريضًا بالعيادات الداخلية في خمسة مستشفيات عامة في أوديشا، وتم بعده تحليل العوامل الاستكشافية. وفي النهاية، قمنا بإجراء مقابلات شخصية مع 15 فرداً لتقييم صلاحية محتوى عناصر المسح.

**النتائج:**

كشف الاختبار الإدراكي أن ستة من أصل 18 سؤالًا من أسئلة المسح لم تكن مفهومة بشكل متسق داخل بيئة المرضى في العيادات الداخليين في أوديشا، مما يركز على المشكلات المتعلقة بمسؤوليات الرعاية. أدى تحليل عوامل الاستكشاف إلى تحديد بنية مكونة من ستة عوامل تشرح %66.7 من التباين. أظهرت نماذج التحوف أن الرعاية بين الأشخاص من الأطباء والممرضات كان لها ارتباط وثيق بالرضا العام. كشف تقييم عمل العناصر المتباينة أن المرضى الذين ينتمون لطبقة مهمشة اجتماعيًا أبلغوا عن رعاية أعلى تخلو من الاحترام، على الرغم من أن هذا لم يُفسّر بأنه اختلافات في الرضا المبلغ عنه. تشير فهرسة صلاحية المحتوى إلى أن الاختلاف بين تجارب الرعاية التي تخلو من الاحترام، وتقييمات الرضا، قد يكون بسبب انخفاض توقعات المرضى.

**الاستنتاج:**

إن استخدام معدلات الرضا دون اتباع أساليب دقيقة في برامج الشراء القائمة على القيمة، قد يؤدي إلى إخفاء الخدمات ذات الجودة الرديئة بين الأشخاص، وخاصة بالنسبة للمرضى المهمشين لفترات طويلة. ويجب تصميم المسوح بحيث تلتقط بدقة المستويات الحقيقية لعدم الرضا، مما يضمن عدم إخفاء مخاوف المرضى.

## 摘要

**目的:**

研究住院患者满意度普查作为医院绩效衡量标准的表现如何。

**方法:**

我们在印度奥里萨邦开展了一项基于混合方法的试点研究，以调查医院消费者对卫生保健提供者和系统的评价情况。我们将研究分为三个步骤：对调查的认知测试、包括探索性因素分析的项目测试和内容效度指数评估。认知测试纳入了 50 名参与者，以讨论他们对调查项目的理解。然后，我们对奥里萨邦五家公立医院的 507 名住院患者进行了调查，随后又进行了探索性因素分析。最后，我们采访对 15 人进行了，以评估调查项目的内容效度。

**结果:**

认知测试结果显示，在针对奥里萨邦住院部的 18 个调查问题中，受访者对其中 6 个问题的理解不一致，这突出了护理责任方面存在的问题。通过探索性因素分析，我们确立了一个，该模型解释存在 66.7% 的方差六因素结构模型。回归模型显示，医生和护士对患者的照护情况与总体满意度的相关性最强。差异项目评估结果显示，属于社会边缘化种姓群体的患者报告受到不尊重照护的情况较多，然而这并非造成所报告满意度方面差异的主要原因。内容效度指数评估表明，受到不尊重照护与满意度评分之间的不一致性可能是由于患者期望值较低造成的。

**结论:**

针对基于价值的购买项目，使用满意度评分而不采用细致入微的评估方法，可能会掩盖低质量的患者照护情况，特别是对历史上属于边缘化群体的患者而言。调查的设计应能保证准确了解真实的不满意程度，从而确保患者的担忧不会被隐藏。

## Резюме

**Цель:**

Изучить, как общий опрос пациентов стационара с целью выявить степень удовлетворенности качеством обслуживания используется в качестве показателя эффективности работы больницы.

**Методы:**

Было проведено поисковое исследование с использованием смешанных методов в рамках опроса потребителей медицинских услуг и систем медицинского обслуживания в больницах, Одиша, Индия. Исследование было разделено на три этапа: когнитивное тестирование опросника, тестирование элементов с помощью эксплораторного факторного анализа и определение валидности содержания. Когнитивное тестирование включало в себя обсуждение 50 участниками своей интерпретации пунктов опросника. Затем опрос был проведен среди 507 стационарных пациентов в пяти государственных больницах штата Одиша, после чего был проведен эксплораторный факторный анализ. Наконец, было проведено интервью с 15 лицами для оценки валидности содержания пунктов опроса.

**Результаты:**

Результаты когнитивного тестирования свидетельствуют о том, что шесть из 18 вопросов анкеты не всегда были понятны в условиях стационара в Одише. Это указывает на проблемы, связанные с ответственностью за уход. В результате эксплораторного факторного анализа была выявлена шестифакторная структура, объясняющая 66,7% дисперсии. Регрессионные модели показали, что межличностная забота со стороны врачей и медсестер наиболее сильно связана с общей удовлетворенностью. Оценка дифференцированного функционирования пунктов показала, что пациенты из социально маргинализированной касты отмечали более неуважительное отношение к себе, хотя это не отражалось на различиях в показателях удовлетворенности. Проверка валидности содержания показала, что несоответствие между впечатлениями от неуважительного отношения и оценками удовлетворенности может быть связано с низкими ожиданиями пациентов.

**Вывод:**

Использование оценок удовлетворенности без учета нюансов в программах закупок, основанных на ценностях, может скрыть низкое качество межличностных услуг, особенно для исторически маргинализированных пациентов. Опросники необходимо разрабатывать таким образом, чтобы точно фиксировать истинный уровень неудовлетворенности, гарантируя, что проблемы пациентов не будут скрыты.

## Introduction

In 2018, the Indian government launched the world’s largest health insurance scheme, *Pradhan Mantri Jan Arogya Yojana*.^1^ The scheme aims to cover secondary and tertiary care for 500 million newly insured citizens, corresponding to 40% of the country’s most vulnerable population.^2^^–^^4^ The government has focused on the quality of care covered through the scheme, including patient satisfaction as a key quality metric in several accountability programmes.^5^^,^^6^ A proposed nationwide programme would formally tie hospital performance to payment with up to 15% of reimbursement depending on the quality of services delivered.^7^ Satisfaction is the programme’s primary proposed measure of patient-centred care, similar to many value-based purchasing programmes in high-income countries that incentivize high-quality care by linking hospital payments to performance.^8^ Hence, poor performance on patient satisfaction measures may represent a substantial financial risk for hospitals.

The Ministry of Health and Family Welfare of India has long prioritized measuring patients’ satisfaction with secondary and tertiary care. For example, *Mera Aspataal* (My Hospital) is a health ministry digital platform used to capture patient feedback on services received from both public and private health facilities.^9^ To develop this platform, the health ministry used a review of validated patient surveys.^6^ *Mera Aspataal* data have informed three policy efforts: a public reporting programme, the national hospital accreditation programme, and a results-based incentives effort focused on hospital cleanliness and physical infrastructure.^6^ Alternate sources of information, such as insurance claims data, on the quality of health services delivered in inpatient settings across India are scarce.^10^^,^^11^ However, the use of patient satisfaction measures within payment programmes has been controversial^8^and there are debates on how best to interpret and value satisfaction ratings.^12^^,^^13^ Implicit in any survey-based measure is the assumption that tools are consistently understood by the patient and that variation represents the underlying construct being assessed, as opposed to differences in how people understand or interpret a concept or tool.^14^ Critics argue that due to information asymmetry, some patients may rate the superficial aspects of the visit (for example, an imposing lobby) rather than the technical or interpersonal quality of care provided by health workers.^15^ This issue may be particularly relevant as low- and middle-income countries improve access to hospital-based care, and newly insured patients may use secondary and tertiary services for the first time.^2^^,^^16^ While the health ministry already prioritizes patient satisfaction, we lack an in-depth understanding of how patients understand and value aspects of the care interaction, and how those understandings inform satisfaction reporting in the context of a value-based purchasing programme.^7^

To better understand how satisfaction ratings function within an Indian inpatient setting, we conducted a pilot study using a comprehensive survey tool that assesses both patients’ experiences with a given clinical interaction and their overall satisfaction rating. Considering the proposed value-based purchasing programme, we posed the following research questions: what aspects of patient experience do patients value when rating their satisfaction with care? Does the tool function similarly across different patient types? What factors might drive differences in reporting and to what extent might they be systematic?

## Methods

We conducted a mixed-methods assessment of a comprehensive patient experience survey tool, focusing on how patients report overall satisfaction with general inpatient care.^7^ We employed methods similar to those used in the development of the tool (Table 1).^17^ We divided the study into three steps: cognitive testing of the survey; item testing and exploratory factor analysis; and content validity indexing. We built on prior work on patient satisfaction in Indian clinical settings.^5^^,^^18^ We used the Hospital Consumer Assessment of Health Providers and Systems survey, due to its use in the nationwide value-based purchasing programme in the United States of America^19^ and its relevance to India’s proposed programme.^7^ The survey includes a direct overall measure of patient satisfaction and has been tested in nine countries worldwide.^20^^–^^24^ In India, the tool and its derivatives have been used to assess hospital quality and inform digital health platforms.^6^ The survey includes questions assessing aspects of the patients’ experience across six domains: interpersonal care from nurses; interpersonal care from doctors; the hospital environment; general experience; after-discharge care; and understanding of care.^25^ These patient experience questions employ a four-point Likert scale, and additional questions collect demographic information, such as age and gender. 

**Table 1:** Methods used to pre-test and pilot the Hospital Consumer Assessment of Health Providers and Systems survey, Odisha, India, 2020

Step^a^	Purpose	Process	No. of participants^b^
1. Cognitive testing	To refine translation of survey tool. To ensure variation in responses do not reflect differences in understanding of a given question, we aimed to identify how individuals interpret each survey item and how their cognitive processing relates to the construct intended by the researcher and original survey instrument	Focus groups discuss all survey items to assess if framing is logical and answerable, if response options are adequate, etc. We paired each item with structured verbal probes to elicit participants’ cognitive processes and assess their understanding and interpretation of each survey item	50
2. Item testing and exploratory factor analysis	Quantitively assess how survey items relate and if exposure to quality of care informs our overall variable of interest: patient satisfaction	Hospital-based exit interviews with eligible patients; responses anonymized and analysed using an exploratory factor analysis and series of ordinary least squares models with overall satisfaction posed as a dependent variable, controlling for patient complexity and interview characteristics, for example privacy and enumerator ID	507
3. Content validity indexing	Assess to which extent the tool items represent facets of the construct patient experience, that is, do the survey items represent what is important to patient-centredness in Odisha, India	One hour-long individual interviews, conducted in non-clinical settings with five patients, five health workers and five health-system experts. For each survey item, each interviewee rates the relevance to patients’ satisfaction and relevance given hospital environment, using a four-point Likert scale. Subsequently, interviewees describe the reasons for their ratings	15

^a^ Steps were conducted consecutively.

^b^ Participants partook only in one step, that is each group was distinct.

### Step 1

To ensure that observed variation reflects real differences and is not the result of heterogeneity in how the questions are interpreted,^14^ we used cognitive testing.^17^^,^^26^^–^^29^ In this assessment, respondents discussed what each survey item meant to them with the goal of exploring the processes by which respondents answer survey questions. We followed the protocol developed for the Hospital Consumer Assessment of Health Providers and Systems survey.^17^ Participants included 50 convenience-sampled Odia-speaking individuals, 27 women and 23 men (gender was self-reported). We conducted the cognitive testing in Bhubaneswar, India, with all assessments in Odia, and clarifying discussions in Odia, Hindi and English. During a day-long session, participants reviewed each survey question in full, working in focus groups of 7 to 12 individuals to discuss their understanding of each question. We reimbursed the individuals for their participation. We used scripted probes to elicit additional insights into cognitive processes and conceptual equivalence in processing survey items.^30^ We used deductive qualitative analysis to categorize identified issue types.

### Step 2

We administered the Odia-translated Hospital Consumer Assessment of Health Providers and Systems survey to patients at the time of discharge who had been hospitalized for at least 24 hours. We sampled five public hospitals across Odisha from purposively selected districts. Districts were first grouped according to administrative units, then selected to represent the diversity of the state in terms of tribal population, urbanization, coastal and mining areas, which are believed to influence health, health-care utilization and health-related expenditure. For each hospital, we surveyed approximately 100 patients (20 female obstetrics inpatients, 40 general female and male inpatients each) with an average survey duration of 35 minutes. When the number of patients being discharged exceeded the number of patients the enumerators were able to survey, we used a stratified random sampling strategy with a list frame approach to reduce bias. We set the target sample to 500 respondents, which exceeds recommendations for quantitative validation involving patients (250–350 patients)^31^ and meets the threshold of very good for factor analysis.^32^

With the resulting survey data, we conducted an exploratory factor analysis using principal-component factors (assuming no unique factors), and calculated the average of all correlations between each item and the total score (Cronbach's *α*). Additionally, we ran three models examining the relationship between individual survey items and overall patient satisfaction. Model I is an unadjusted bivariate ordinary least squares regression where overall satisfaction is the dependent variable, and each patient experience survey item is treated as a separate independent variable. Model II adds the patient’s age and gender, as well as variables relevant to clinical complexity: if the patient was admitted through the emergency department; the patient’s self-reported rating of health; length of stay; and facility type. Model III adds variables relevant to the interview: interviewer ID and an enumerator rating of interview privacy. Finally, we assessed differential item functioning by disaggregating results by caste, assessing differences in means with a two-sample *t*-test, and producing a Spearman’s rank correlation coefficient for each subgroup to assess the strength of the relationship between exposure to disrespectful care and odds of reporting dissatisfaction. Dissatisfaction is shown as an unweighted proportion, with the four most negative response options (of 10) combined to generate one negative rating.

### Step 3

To assess the degree to which questionnaire items constitute an adequate operational definition of our construct of interest,^33^ that is, patients’ overall satisfaction, we used item-level content validity indexing.^21^ We interviewed 15 individuals, purposively sampled across three categories – patients, health workers and experts. Patients were people familiar with public hospital care in Odisha and included hospital patients on the day of discharge; health workers were currently providing clinical care in Odisha; and experts were researchers experienced in collecting patient data from inpatient settings in Odisha. Each interview was in-person and lasted approximately one hour. The interviews involved providing verbal instructions on how to use the Likert scale (1: not relevant; 2: somewhat relevant; 3: relevant; and 4: highly relevant) to evaluate the relevance of survey items, followed by questions to explain why they did, or did not, think the item was relevant. Two separate scores were captured: (i) the item’s relevance to patient satisfaction; and (ii) the item’s relevance given the clinical setting. By allowing interviewees to provide two distinct scores, we were able to address concerns regarding care expectations identified during cognitive testing. This approach helped us better distinguish whether low ratings were due to concerns with the item’s relevance to patient satisfaction, or other factors, such as feasibility and structural constraints in the study setting.

### Disaggregating expectations

Finally, to outline policy-relevant implications of this work, we used Thompson and Sunol’s framework to organize sources of variation into four categories: ideal expectations, predicted expectations, normative expectations and patient expression.^34^

### Ethical considerations

Institutional Review Board approval was provided through Harvard TH Chan School of Public Health, Boston, United States of America (IRB18–1675); Research and Ethics Committee of the Directorate of Health Services, Government of Odisha ID: 60/PMU/187/17; and Sigma, registered with the Division of Assurance and Quality Improvement of the Office for Human Research Protections, USA (IRB00009900). All participants gave informed consent to participate in the study before taking part.

## Results

Participants in the cognitive testing surfaced several fundamental concerns. They flagged six out of 18 questions as having relevance issues to the Odisha inpatient setting. These issues centred around responsibility for care. For example, families, not health workers, may be responsible for cleanliness. Furthermore, participants thought that doctors were responsible for communicating clinical information, but did not think they were responsible for explaining the information. These concerns informed conversations about which tasks were the responsibilities of health-care professionals (Table 2).

**Table 2:** Cognitive testing issues identified in items in the Hospital Consumer Assessment of Health Providers and Systems survey, Odisha, India, 2020

Survey domain and item	Full item text	Cognitive testing issue	
		Brief description	Type^a^
**Interpersonal care from nurses**			
Courtesy and respect	During this hospital stay, how often did nurses treat you with courtesy and respect?	No issues raised	NA
Listen carefully	During this hospital stay, how often did nurses listen carefully to you?	Listening carefully may not be seen as distinct from being treated with respect	Construct
Explain	During this hospital stay, how often did nurses explain things in a way you could understand?	Patient must define “how often,” as the concept often lacks a point of reference	Construct
**Interpersonal care from doctors**			
Courtesy and respect	During this hospital stay, how often did doctors treat you with courtesy and respect?	No issues raised	NA
Listen carefully	During this hospital stay, how often did doctors listen carefully to you?	Doctors are often not responsible for listening to patients	Relevance
Explain	During this hospital stay, how often did doctors explain things in a way you could understand?	Doctors are often not responsible for explaining care to patients	Relevance
**Hospital environment**			
Room clean	During this hospital stay, how often were your room or ward and bathroom kept clean?	Families, not providers, are often responsible for cleanliness	Relevance
Quiet	During this hospital stay, how often was the area around your room/ward quiet at night?	Lack of clarity on the concept quiet. In open hospital wards, it may not be possible to maintain quiet	Construct and relevance
**General experience**			
Bathroom help	How often did you get help in getting to the bathroom or in using a bedpan as soon as you wanted?	Families, not providers, are often responsible for bedpans	Relevance
Talk about pain	During this hospital stay, how often did hospital staff talk with you about how much pain you had?	Patient must define “how often,” as the concept often lacks a point of reference	Construct
Talk about pain treatment	During this hospital stay, how often did hospital staff talk with you about how to treat your pain?	Patient must define “how often,” as the concept often lacks a point of reference	Construct
Explain medication purpose	Before giving you any new medicine, how often did hospital staff tell you what the medicine was for?	Lack of clarity on what constitutes new medicine. External purchase of medication most common and doctors rarely provides the medicine	Information and relevance
Explain side-effects of medication	Before giving you any new medicine, how often did hospital staff describe possible side-effects in a way you could understand?	Lack of clarity on what constitutes new medicine. External purchase of medication most common and doctors rarely provides the medicine	Information and relevance
**After discharge**			
Assessment of post-discharge	During this hospital stay, did doctors, nurses or other hospital staff talk with you about whether you would have the help you needed when you left the hospital?	Understood as: when you go home will you get the help that you need	Construct
Receipt of discharge guidance	During this hospital stay, did you get information in writing about what symptoms or health problems to look out for after you left the hospital?	Written guidance may be irrelevant if patients are illiterate	Relevance
**Understanding of care**			
Taking preferences seriously	During this hospital stay, staff took my preferences and those of my family or caregiver into account in deciding what my health care needs would be when I left.	The doctors may not concern themselves with care after discharge, as it is not within the scope of the doctor’s professional role	Relevance
Understand responsibilities	When I left the hospital, I had a good understanding of the things I was responsible for in managing my health.	Lack of clarity on what the patient is told versus what the patient understands	Construct
Understand purpose of medications	When I left the hospital, I clearly understood the purpose for taking each of my medications?	No issues raised	NA

NA: not applicable

^a^ Construct issues were raised when the item was understood differently than its intended construct. Information issues were raised when there was unclear or inadequate information for a patient to answer the question reliably. Relevance issues were when there was something about the question that raised concern, e.g. relevance in the Odisha inpatient setting.

In step 2, enumerators surveyed 507 patients. Educational backgrounds varied, with most male inpatients having completed a primary or middle school education (77/193), while most female inpatients had no formal schooling (62/209). The majority identified as Hindu (494/507) and most spoke Odia (442/507) as their primary language (Table 3).

**Table 3:** Characteristics of public hospital-based exit interviewees, Odisha, India, 2020

Characteristic	No. of respondents (%)^a^		
	Male inpatients (*n* = 193)	Female inpatients (*n* = 209)	Inpatients of obstetrics–gynaecology departments (*n* = 105)
**Age in years, mean (SD)**	47.2 (17.6)	45.2 (17.4)	25.5 (5.3)
**Highest educational attainment**			
Illiterate	13 (6.7)	32 (15.3)	0 (0.0)
No formal schooling	32 (16.6)	62 (29.7)	11 (10.5)
Under primary	11 (5.7)	22 (10.5)	13 (12.4)
Primary	39 (20.2)	21 (10.1)	15 (14.3)
Upper primary and middle	38 (19.7)	24 (11.5)	18 (17.1)
Secondary	29 (15.0)	25 (12.0)	23 (21.9)
Higher secondary	19 (9.8)	13 (6.2)	21 (20.0)
Graduate	7 (3.6)	7 (3.4)	4 (3.8)
**Caste**			
Scheduled tribe	34 (17.6)	40 (19.1)	28 (26.7)
Scheduled caste	23 (11.9)	36 (17.2)	25 (23.8)
Otherwise backward class	74 (38.3)	64 (30.6)	22 (20.9)
General^b^	61 (31.6)	67 (32.1)	29 (27.6)
**Religion**			
Hindu	189 (97.9)	205 (98.1)	100 (95.2)
Muslim	4 (2.1)	4 (1.9)	1 (1.0)
Christian	0 (0.0)	0 (0.0)	4 (3.8)
**Primary language^c^**			
Odia	171 (88.6)	193 (92.3)	78 (74.3)
Hindi	4 (2.1)	4 (1.9)	1 (1.0)
Telugu	0 (0.0)	2 (1.0)	3 (2.9)
Tribal dialect	16 (8.3)	9 (4.3)	21 (20.0)

SD: standard deviation.

^a^ Values are no. (%) if not otherwise given.

^b^ No historically marginalized caste designation.

^c^ Languages spoken by less than 1% of respondents not included, hence the sum does not equal 100%.

Note: we limited the sampling to public hospitals which are slated to be incorporated within the proposed value-based purchasing programme.

The exploratory factor analysis yielded six eigenvalues greater than 1, indicating a six-factor structure. These results explained 66.7% of the variance within the model. All Cronbach’s *α* values exceeded the threshold of 0.7. Uniqueness at the item-level, variance not shared with other variables, ranged from 17.1% (understand responsibilities) to 55.6% (doctors listen carefully). Regression models revealed that the hospital environment category had the weakest association with overall satisfaction (Model III coefficient: 0.23), whereas interpersonal care from doctors and nurses had the strongest association (Model III coefficients: 0.76 and 0.70, respectively; Table 4).

**Table 4:** Results of exploratory factor analysis and of overall satisfaction models, Odisha, India, 2020

Category and experience item	Mean item value (SE)	Exploratory factor analysis and item-level testing			Coefficient, by level							
					Model I^b^			Model II^c^			Model III^d^	
		Item uniqueness	Cronbach’s *α*^a^		Item	Category		Item	Category		Item	Category
**Interpersonal care from nurses (λ: 3.5)^e^**												
Courtesy and respect	3.4 (0.034)	0.221	0.785		0.65***	0.76***		0.58***	0.69***		0.59***	0.70***
Listen carefully	3.4 (0.032)	0.218	0.781		0.79***			0.74***			0.75***	
Explain	3.3 (0.036)	0.371	0.780		0.81***			0.75***			0.77***	
**Interpersonal care from doctors (λ: 1.9)^e^**												
Courtesy and respect	3.5 (0.031)	0.359	0.785		0.91***	0.82***		0.84***	0.74***		0.86***	0.76***
Listen carefully	3.3 (0.033)	0.556	0.779		0.82***			0.73***			0.75***	
Explain	3.3 (0.033)	0.319	0.785		0.72***			0.65***			0.66***	
**Hospital environment (λ: 1.7)^e^**												
Room clean	2.9 (0.040)	0.293	0.798		0.47***	0.33***		0.39***	0.25**		0.38***	0.23**
Quiet	2.5 (0.044)	0.287	0.807		0.18***			0.10			0.08	
**General experience (λ: 1.3)^e^**												
Talk about pain	2.6 (0.056)	0.445	0.790		0.90***	0.68***		0.81***	0.60***		0.87***	0.62***
Talk about pain treatment	2.9 (0.036)	0.310	0.786		0.64***			0.56***			0.57***	
Explain medication purpose	2.8 (0.055)	0.330	0.802		0.50***			0.42***			0.42***	
**After discharge (λ: 1.3)^e^**												
Assessment of post-discharge	0.9 (0.022)	0.345	0.811		0.26*	0.54**		0.09	0.34**		0.09	0.33**
Receipt discharge guidance	1.6 (0.017)	0.542	0.801		0.81***			0.59***			0.57***	
**Understanding of care (λ: 1.1)^e^**												
Taking preferences seriously	3.6 (0.024)	0.300	0.804		0.69***	0.69***		0.55***	0.58***		0.54***	0.57***
Understand responsibilities	3.6 (0.023)	0.171	0.801		0.72***			0.59***			0.58***	
Understand purpose of medications	3.6 (0.026)	0.277	0.804		0.67***			0.59***			0.58***	

SE: standard error; *P ≤ 0.05; **P ≤ 0.01; ***P ≤ 0.001.

^a^ A typical exclusion threshold for *α* coefficient is 0.70. The higher the *α* coefficient, the more the items have shared covariance and may measure the same underlying concept. Highly correlated items will also produce a high coefficient and can therefore be interpreted as a sign of redundancy. As we did not conduct the analysis to shorten the Hospital Consumer Assessment of Health Providers and Systems survey, we retain all items regardless of performance.

^b^ Model I represents the unadjusted results of a bivariate ordinary least square regression where overall satisfaction is the dependent variable and each row represents a different patient experience item posed to patient.

^c^ Adjusted for patient age, gender and clinical complexity.

^d^ Adjusted for Model II factors plus interview characteristics.

^e^ Eigenvalues (λ) shown for retained factors. Corresponding item categories are discrete and align with factor loadings most relevant to defining each factor’s dimensionality.

Note: we excluded two items (bathroom help and explanation of medicine side-effects) from this table because fewer than 50 respondents needed support with the bathroom or were prescribed medicines.

**Fig. 1:**
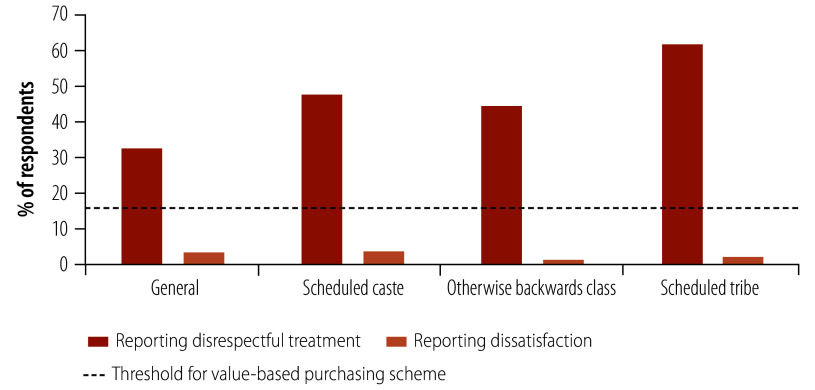
Share of patients reporting receipt of disrespectful treatment and share reporting overall dissatisfaction with care, by caste, Odisha, India, 2020

Notes: the proposed value-based purchasing programme in India sets an initial threshold of 85% satisfaction (15% dissatisfaction). We combined the four most negative response options (of 10) to generate a combined negative rating. We used this interpretation of dissatisfaction because the satisfaction ratings in India’s proposed value-based purchasing programme will be evaluated using a 5-point Likert scale of which the two least favourable responses will be combined to a negative rating. Difference is assessed with a two-sided *t*-test comparing to the base group, individuals with no historically marginalized designation.

Disaggregating results by patient characteristics, we identified differential functioning of survey items based on caste. Patients who identified as part of a scheduled caste, otherwise backward class or scheduled tribe were significantly more likely to report receiving disrespectful care compared to patients with no marginalized class designation (*P*-value: > 0.05; Fig. 1; Table 5). In contrast, there was no statistical difference in reporting dissatisfaction between the groups. Only patients who identified as part of an otherwise backward class had a significant correlation between exposure to disrespectful care and reporting dissatisfaction (*ρ*: 0.19; *P*-value: 0.02). Moreover, all values fall well below the 15% satisfaction threshold set within the proposed value-based purchasing programme, meaning the difference in exposure to disrespectful care by caste would not translate to a difference in hospital payment. 

**Table 5:** Share of patients reporting receipt of disrespectful treatment and share reporting overall dissatisfaction with care, by caste, Odisha, India, 2020

Caste group	Reporting disrespectful treatment	Reporting dissatisfaction	Spearman’s *ρ*^a^ (*P*)
**General^b^ (*n* = 157)**			0.34 (< 0.01)
% of respondents (no.)	32.5 (51)	3.2 (5)	
**Scheduled caste (*n* = 84)**			0.14 (0.19)
% of respondents (no.)	47.6 (40)	3.6 (3)	
Difference from general group, % points (*P*)	15.1 (< 0.01)	0.4	
**Otherwise backward class (*n* = 160)**			0.19 (0.02)
% of respondents (no.)	44.4 (71)	1.3 (2)	
Difference from general group, % points (*P*)	11.9 (0.01)	−1.9	
**Scheduled tribe (*n* = 102)**			0.17 (0.09)
% of respondents (no.)	61.8 (63)	2.0 (2)	
Difference from general group, % points (*P*)	29.3 (< 0.01)	−1.2	

^a^ Spearman’s *ρ* assessing the relationship between reporting disrespectful treatment and reporting dissatisfaction.

^b^ The general group refers to individuals with no historically marginalized class designation.

**Fig. 2:**
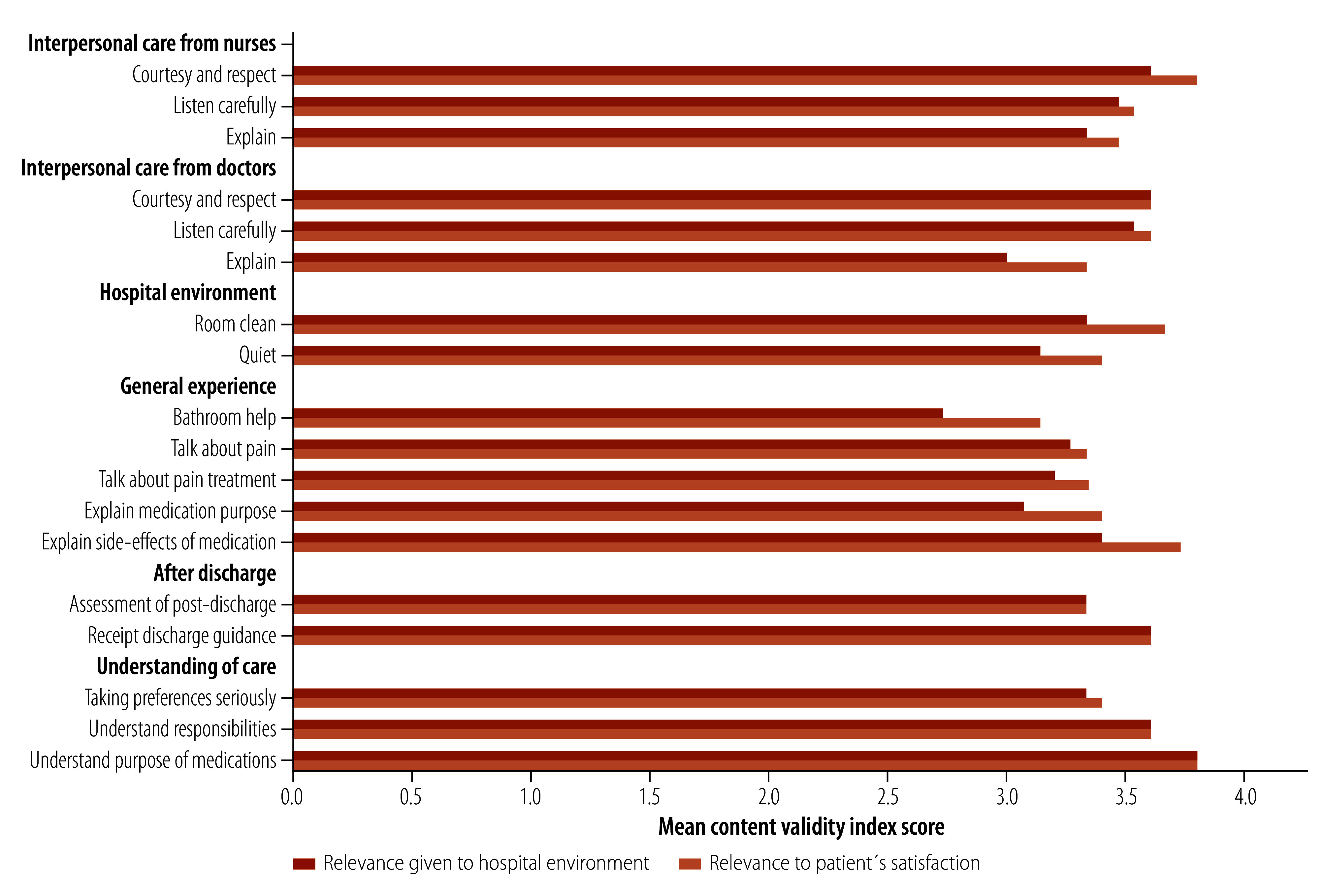
Mean content validity indexing scores assessing items’ relevance to patient satisfaction and hospital environment, Odisha, India, 2020

Notes: we interviewed five patients, five health workers and five health-system researchers, who rated the relevance of survey item to either patient satisfaction or the hospital environment, including feasibility or likelihood of an event occurring in the inpatient setting. Rating scale for each individual question was 1: not relevant; 2: somewhat relevant; 3: relevant; 4: highly relevant.

Finally, our content validity indexing results suggest that reporting discordance (that is, experiencing disrespectful care but not reporting dissatisfaction) may be due to low expectations rather than a difference in what patients value. When participants were asked about item relevance, hospital environment relevance scored lower (Fig. 2) than relevance to patients’ satisfaction in 13 of 18 questions. These results align with cognitive testing results; for example, participants valued doctors listening carefully, but did not expect this to occur in practice because they did not believe it was a physician’s responsibility within the Odisha inpatient setting. 

Interviews revealed that understandings of clinical responsibilities and corresponding expectations informed patients’ overall ratings. For example, a patient participant stated:

“I do feel the doctors were disrespectful, but they are the boss and this is how it is, no? So I think disrespect is important to me and my family, but if this is the same treatment I got last time, why complain? This is why my [satisfaction] score is still high.”

These pilot study findings raise concerns regarding the use of an overall satisfaction rating within provider payment programmes and how we interpret traditional quantitative approaches to validation, which may assume low item functioning means low importance to the patient or satisfaction. Potential sources of variation in patient satisfaction ratings and considerations for value-based purchasing policies are presented in Table 6. These sources suggest a need to consider predicted expectations in addition to other sources of variation. 

**Table 6:** Sources of variation in patient satisfaction ratings and considerations for value-based purchasing policies, Odisha, India, 2020

Source of variation	Description^a^	Policy considerations for value-based purchasing
Values	Ideal expectations are similar to aspirations, desires or preferred outcomes; what a person ultimately values, that is, in a situation without limitation	Values can, and likely do, vary between patients and contexts; expectations represent an anticipated source of variation, allowing satisfaction ratings to reflect a diverse range of patient values
Expectations	Predicted expectations are realistic, practical or anticipated outcomes that result from personal experiences, reported experiences of others and sources of knowledge such as the media	Addressing variation that results from differences in predicted expectations may include the following: - Collecting basic demographic information about patients that are potentially associated with historical marginalization, for example, religious identity, caste and educational attainment. These data can be used to better understand hospitals’ baseline population as well as augment clinically-focused risk adjustment, which is often used within value-based purchasing programmes and focuses on case mix, i.e. morbidity type and severity
	Normative expectations are based on what should or ought to happen, often based on a mutually agreed upon threshold for what constitutes patient-centred care (similar to human rights standards)	Addressing variation that results from differences in normative expectations may include the following: - Pair subjective satisfaction ratings with more objective assessments of what a patient is experiencing during a given clinical interaction (that align with normative guidance) and look for discordance in patient ratings, that is, when patients give positive ratings to potentially inadequate care^b^ - Due to low and variable thresholds for reporting dissatisfaction when exposed to low quality care, do not use a satisfaction rating to trigger sub-items, which are sometimes only posed to dissatisfied patients
Expression	Expression is how patients convey or report their satisfaction with care to others, which may differ for patients regardless of ideal, predicted, or normative expectations of care and inform reporting bias,^c^ that is, how satisfaction is expressed may differ among patients with a similar level of true satisfaction	Addressing variation that results from differences in expression may include the following: - Consider the addition of variables within surveys used for value-based purchasing that may inform reporting bias. For example, interview privacy and interviewer ID. Consider these factors when analysing data to address underreporting, which may be more prevalent for marginalized patients. - If resources allow, follow up with a random subset of interviewed patients to assess if there is a variation in responses once they left the hospital

^a^ Adapted from Thomson & Sunol, 1995.^34^

^b^ For example, being yelled at by a provider is generally seen as unacceptable by both national and international standards. It is important to understand if patients consistently give positive feedback to such care, as this helps ensure that these forms of poor-quality care are challenged, particularly among marginalized patients.

^c^ Thomson & Sunol^34^ include a related concept, which they call “unformed expectations,” which is when individuals are unable to articulate their expectations because they do not have expectations, have difficulty expressing their expectations or do not wish to reveal their expectations due to fear, anxiety or conforming to social norms.

## Discussion

In this pilot study, we find aspects of the care interaction beyond the physical environment, such as the quality of interpersonal care, had a strong relationship with overall satisfaction. However, these results raise concerns for the use of satisfaction ratings within a nationwide performance policy. Observed differences in care ratings may not reflect true differences in patients’ satisfaction, which may vary between sociocultural groups. These findings are timely as the Indian government considers using satisfaction ratings to hold hospitals accountable to patients.

Satisfaction ratings, as a single metric, are appealing in that they theoretically capture a wide range of underlying preferences. Conversely, absent of clinical expertise, patients may place undue value on more superficial aspects of the care interaction – aspects more subject to manipulation to improve ratings.^35^ Contrary to this concern, we found the physical environment had a weak relationship with satisfaction. Patients did appear to value interpersonal aspects of care, for example, being listened to carefully and having care explained adequately. Even when examining questions that did not perform well in the factor analysis or regression models, such as receipt of post-discharge guidance, content validity indexing suggested this guidance was valued, but participants did not anticipate it to occur in practice. Traditionally, in tool validation studies, low item performance in quantitative approaches indicates that the item is not an important driver of patient satisfaction. As a result, the item may be excluded. However, our results indicate that low coefficients may result from low predicted expectations rather than low ideal expectations.

The proposed value-based purchasing programme sets an 85% satisfaction rating threshold, with facilities scoring below facing reduced health insurance scheme reimbursement.^7^ In our study, despite a high proportion of respondents reporting disrespectful care, reimbursement would not be affected since dissatisfaction ratings fell well below 15%. As such, the currently designed programme may not adequately surface low-quality interpersonal care provided to marginalized patients. This type of variation in reporting, which results from differences in predicted expectations, is problematic particularly if certain patients or groups of patients have been systematically subjected to lower quality of care than others. Different thresholds for reporting satisfaction raise concern for the use of overall ratings within value-based purchasing.^36^ Many public reporting and payment programmes treat satisfaction as a stand-alone measure, which is both a feasible and simple approach, particularly if variation results from differences in ideal expectations. However, this approach may fail to surface low-quality interpersonal care experienced by individuals unlikely to report overall dissatisfaction – either due to low predicted expectations or issues of expression. Scheduled tribe patients, for example, may have lower expectations of the system due to experiences of disrespect. Furthermore, patients with higher education may have unreasonable predicted expectations of the health system and/or a lower threshold for the expression of dissatification.^37^ Researchers developing the World Health Surveys coined the term universally legitimate expectations, which refers to a normative set of expectations.^37^ Accordingly, we provide actionable considerations for improving satisfaction ratings within value-based purchasing programmes (Table 6).

This work extends the existing literature assessing patient experience and satisfaction in Indian clinical settings.^5^^,^^38^^,^^39^ We build on this work by focusing on general inpatient care, instead of specific conditions or specialties, and consider policy applications given the proposed value-based purchasing programme. While some studies have used the Hospital Consumer Assessment of Health Providers and Systems tool in India as an outcome measure,^40^ we were unable to find any documentation of formal adaptation or pre-testing processes that might be useful in informing the tool’s use in payment policies. Our work also extends the patient vignette literature, which aims to understand differences in how individuals judge care for a fixed clinical example.^41^^,^^42^ This literature exposes differences in ratings based on patient characteristics, but cannot disentangle why ratings differ. By using a formative mixed-methods approach, we were able to assess patients’ values and expectations.

This study has several limitations. First, the sample size is small and we lacked a reliable sampling frame. For example, due to the small sample, we were unable to examine how patient characteristics interact with one another. However, the results and concerns raised should inform larger studies. Second, we conducted this pilot study in a rural state with a large tribal population, which may pose challenges to generalizing these findings. However, researchers have estimated that the largest increases in hospital utilization will likely occur in states like Odisha, and we lack research on survey tools that assess health system performance in the state.^43^ Third, the study was run as a hospital exit interview as opposed to a non-hospital-based setting, which is considered best practice in mitigating reporting bias.^44^^–^^46^ For example, the likelihood of reporting disrespectful or abusive delivery of care in the United Republic of Tanzania increased nearly 10 percentage points in a post-discharge survey compared to an exit interview.^47^ However, almost half of the women in our study had at most a primary school education, which made the enumerators administer the tool verbally. In addition, only 82.1% (416/507) of patients could provide a phone number and for 70.0% (291/416) of them, the phone belonged to a family member or neighbour. These findings reaffirmed the reliance on exit interviews as the most practical method. The limitation of using an exit interview tool motivated us to adjust for interview characteristics in one of our regression models. Finally, the sample sizes for the cognitive testing and content validity indexing are small and not necessarily representative of the final populations that would be surveyed. In our study, the sample sizes exceeded those published in the pre-testing of the Hospital Consumer Assessment of Health Providers and Systems tool in 2005 (cognitive testing: 41 versus 50 participants; and content validity indexing: 12 versus 15 participants).

In conclusion, increased access to health care does not always guarantee better health outcomes,^48^ potentially due to low-quality services.^49^ Therefore, improving the quality of care is crucial, but measuring it can be challenging. Patient-reported measures offer a promising opportunity for assessment. However, without a nuanced approach to identify sources of systematic reporting error, using satisfaction ratings within value-based purchasing programmes may obscure poor-quality interpersonal care for marginalized patient populations.
